# Is Goitre an Infective Disease?

**Published:** 1916-03-18

**Authors:** 


### IS GOITRE AN INFECTIVE DISEASE?

### A Revolutionary Hypothesis and its Consequences.

The well-known work of Major McCarrison, of the Indian Medical Service, in recognition of which the Royal College of Physicians passed the special degree necessary to raise him in absence to the status of a Fellow in that learned Society, tended to prove that the special forms of goitre common in certain particular parts of Northern India—Gilgit, for example—are due to an infective agent of some kind obtaining access to the individual through the water supply. The special merit of the experiments and the reasoning upon which this conclusion was based was the novelty of the conception: Major McCarrison, in fact, had broken new ground in his researches. In England he has found an able disciple in Mr. Rupert Farrant, a Westminster Hospital worker, who has tested the matter as it affects goitre in this country, and has probed it even deeper than Major McCarrison had done. Mr. Farrant has published several papers recently describing his observations and his hypotheses founded on them. A year or two ago one of them appeared in the *British Medical Journal*,* and summarised, in convenient form, the chief points of his position.

### Goitre as a Coli Toxæmia.

Endemic goitre, according to the researches and deductions of Mr. Farrant, is caused by the toxins from atypical forms of *bacillus coli*. This dogma may appear startling when it is reflected that *bacillus coli* is a normal inhabitant of the intestinal canal of everyone; but Mr. Farrant arrays a mass of evidence in support of it, and it will be noted that he has a saving clause in the adjective "atypical." As in McCarrison's researches, water is blamed as the vehicle of contagion. Thus conveyed into the interior economy of the individual, the atypical microbes become indigenous and can be found in the fæces of cases of endemic goitre. They are but seldom found in the fæces of normal individuals, or in those of individuals goitrous from other causes (*e.g.*, from cancer or other new growth).

It is possible to conceive, continues Mr. Farrant, circumstances which place the *bacillus coli* under abnormal conditions in the intestine itself, and lead to mutation and the temporary appearance of mutants in the fæces of normal individuals. In other words, the change from a normal to an abnormal variety of *bacillus coli* might take place inside the body instead of outside it. At all events, the "mutants" (atypical bacilli), however they gain access to the intestines, set up an apyrexial toxæmia which stimulates the thyroid, so leading to a colloid hyperplasia and eventually to enlargement of the gland. The whole process can be initiated in the laboratory, and endemic goitre can be induced in guinea-pigs by feeding them with small doses of the mutants. The supervention of a fresh toxæmia whilst the gland is in a hyperactive state causes a complete hyperplasia with absorption of colloid and signs of hyperthyroidism up to a condition of exophthalmic goitre. This is dependent on the intensity and duration of the fresh toxæmia. According to this view, it will be perceived, exophthalmic goitre is only an advanced stage of the ordinary parenchymatous goitre so common in the population. Those who have followed the progress of research in respect of an allied organism, the *bacillus typhosus*, will not overlook the fact that there is a faint analogy between the hypothetical mutations of the colon bacillus and the existence of the now well-recognised paratyphoid organisms.

Endemic goitre is preventable by the avoidance of water contamination and by the sterilisation of contaminated water. It can be cured by the administration of intestinal antiseptics, and the gland will return to normal providing no degeneration has taken place. The gland as a whole involutes to normal, but the adenomata or cysts are left. A condition similar to endemic goitre can be caused by other toxæmias capable of inducing a colloid hyperplasia.

### Exophthalmic Goitre.

Exophthalmic goitre is due to a combination of toxæmias of an intensity sufficient to cause a hyperplasia with absorption of the colloid material. One acts during a period sufficient to give rise to a complete hyperplasia, associated perhaps with slightly marked signs of hyperthyroidism without necessarily any glandular enlargement. The supervention of another infection stimulates the gland, which usually enlarges, and the signs of hyperthyroidism become very evident: the case develops into one of typical exophthalmic goitre. A nervous shock may lead to the diagnosis by suddenly bringing into evidence the symptoms of hyperthyroidism, especially those connected with the nervous system. The severity and duration of exophthalmic goitre are dependent on the intensity and duration of the toxæmias. If they be of short duration the disease will disappear in a few months.

Exophthalmic goitre can be prevented by the detection of the early cases of hyperthyroidism and the consequent removal of the basal toxæmia. Exophthalmic goitre can be cured if the causatory agents be removed before degeneration has occurred either in the gland or in those organs that are affected by the hypersecretion.

When degeneration has taken place in the thyroid, removal of the toxæmia causes involution to take place only in the hypertrophied portion. Adenomata and cysts are left, and require appropriate surgical treatment, as they keep up to a certain extent the symptoms of thyroid excess. Surgical treatment without removal of the cause is followed by recurrence unless so much of the gland substance has been removed that hypersecretion is impossible. Degeneration in the other organs

* July 18, 1914, p. 110.

partially recovers after involution of the thyroid: appropriate treatment is necessary for those that remain. Acute cases of exophthalmic goitre may present themselves in which surgical treatment is the only hope of saving life, though the risking of death under the anæsthetic may perhaps preclude operation.

It will be seen that the pronouncements are somewhat full of vague terminology and hypothetical presumptions. This criticism is unquestionably necessary, and a great deal more work will certainly be required before this intestinal theory of goitre will be acceptable. This is not to say that Mr. Farrant is on a false scent: we should be very sorry indeed to suggest any such impression, for the ingenuity no less than the sincerity of his work is manifest throughout it. It is quite possible that a considerable foundation of truth underlies the superstructure he has reared upon it, and that at some future time his views, or some subsequent modification of them, may be generally accepted as scientifically substantiated. At the moment this cannot be said. His theories of goitre are fascinating, and must be carefully investigated. But they are not at present anything more than hypothetical, and his able advocacy must not be allowed to obscure this essential fact. It is legitimate, however, to congratulate British medicine on the possession of two such original and brilliant torch-bearers in this particular pathway of advance as Major McCarrison and Mr. Farrant. Further than that it is unsafe at present to pursue the subject, though undoubtedly in a few years this scientific timidity will be unnecessary and out of date.

